# The Peculiar Estrogenicity of Diethyl Phthalate: Modulation of Estrogen Receptor α Activities in the Proliferation of Breast Cancer Cells

**DOI:** 10.3390/toxics9100237

**Published:** 2021-09-25

**Authors:** Marco Fiocchetti, Giovanna Bastari, Manuela Cipolletti, Stefano Leone, Filippo Acconcia, Maria Marino

**Affiliations:** Department of Science, University Roma Tre, Viale G. Marconi, 446, 00146 Rome, Italy; gio.bastari@stud.uniroma3.it (G.B.); manuela.cipolletti@uniroma3.it (M.C.); stefano.leone@uniroma3.it (S.L.); filippo.acconcia@uniroma3.it (F.A.)

**Keywords:** diethyl phthalate, endocrine disruptors, estrogen receptor α, signal transduction pathways, breast cancer cell proliferation

## Abstract

Phthalates comprise a group of synthetic chemicals present in the environment because of their wide use as plasticizers and as additives in products for personal care. Among others, diethyl phthalate (DEP) is largely used in products for infants, children, and adults, in which its exposure has been correlated with an increased risk of breast cancer. The adverse health outcomes deriving from phthalate exposure have been associated with their activity as endocrine disruptors (EDCs) of the steroid and thyroid hormone signaling by affecting developmental and reproductive health, and even carcinogenicity. However, the estrogen disruptor activities of DEP are still controversial, and the mechanism at the root of the estrogenic-disrupting action of DEP remains to be clarified. Here, we evaluated the DEP mechanism of action on the activation status of estrogen receptor α (ERα) by analyzing the receptor’s phosphorylation as well as both nuclear and extra-nuclear pathways triggered by the receptor to modulate the proliferation of breast cancer cells. Although DEP does not bind to ERα, our results suggest that this phthalate ester exerts multiple parallel interactions with ERα signaling and emphasize the importance to determine an appropriate battery of in vitro methods that will include specific molecular mechanisms involved in the endocrine disruption.

## 1. Introduction

The most active form of estrogen, 17β-estradiol (E2), regulates secondary sex characteristics and gametogenesis in the female reproductive system and exerts profound effects in terms of the balance between cell differentiation and growth, also playing a role in non-reproductive tissues [1,2,3]. Moreover, E2 plays an important role in male reproduction and fertility [4]. Two members of the nuclear receptor superfamily, namely, estrogen receptor α (ERα) and β (ERβ), represent the main mediators of E2 pleiotropic effects by acting as ligand-activated transcription factors of genes containing estrogen response element (ERE) sequences [1,5]. Besides the classical direct nuclear action, the engagement of membrane-tethered ERs triggers the activation of several rapid extra-nuclear signaling pathways, which integrate at different levels with the nuclear ones in the definition of E2 physiological functions [1,5,6,7,8,9]. As the critical role of E2 and its cognate receptors in human physiology, any event dysregulating the estrogenic pathway, including receptor subtype balance and their mechanisms of action, can deeply interfere with the physiological development and function of certain organs, potentially inducing reproductive disorders and cancers [10]. 

According to the U.S. Environmental Protection Agency (EPA), endocrine disruptor chemicals (EDCs) are chemical agents, mostly human-made, that interfere with the synthesis, secretion, transport, binding, or elimination of natural hormones responsible for homeostasis maintenance, reproduction, development, and/or behavior [11,12]. Estrogenic EDCs have been studied for a long time, but it is debatable whether most of the EDCs are able to bind to ERs, exerting a mimetic or anti-estrogenic effect on receptor transcriptional activities and, thus, altering a wide range of signaling processes and cellular functions [3,13,14]. The group of estrogenic EDCs also includes phthalates. 

Phthalates (esters of phthalic acid) are chemical compounds primarily used as plasticizers, and many consumer products including building materials, medical equipment, household furnishings, cosmetics, food, beverages, clothes, and toys contain specific members of this chemical family [15,16,17]. Among phthalates, exposure to diethyl phthalate (DEP) is more common for humans, being predominantly connected with the usage of personal care products including perfumes, shampoo, cosmetics, and detergents [15,18,19]. Phthalate exposure through oral, dermal, and inhalation contact has been associated with developmental and reproductive abnormalities including sperm damage, early onset of puberty, infertility, alteration of the reproductive tract, and adverse effects on pregnancy ([20] and reported citations). Such observations have established these compounds as suspected endocrine disruptors [16,21], which are thought to interfere with steroid hormone (e.g., E2 and testosterone) biosynthesis pathways and androgen receptor (AR), thyroid receptor (TR), and ERα signaling [20,21]. Notwithstanding, for a long time, dermal exposure to DEP has been considered to be safe, not presenting a significant toxic liability [19]. However, DEP exposure has been correlated with a 2-fold increase in breast cancer risk [22], raising questions about the possible detrimental effect of DEP at non-toxic doses which can occur through a hormonal disruption mechanism. Although in vivo and in vitro evidence has suggested an estrogenic nature of DEP [18,23,24], other reports [16,19,25] challenged the estrogenic potential of DEP through direct estradiol mimicking potency or even generally, indicating that the estrogenic-disrupting mechanisms of DEP are still a matter of controversy. 

Despite substantial advances in understanding EDC effects and their mechanisms of action, the screening and definition of estrogenic interference have been historically focused on their ability to modulate the DNA binding and direct transcriptional effect of estrogen receptors through the classical nuclear mode of action [5,26]. Extra-nuclear effects have been commonly neglected in the study of EDC estrogenicity, leading to (i) a substantial underestimation of the potential pool of environmental xenoestrogens and (ii) creating a real challenge in the evaluation of EDC risk assessment in the regulatory context. 

In the present study, the estrogenic potential of DEP was evaluated, expanding the “classical” methodological path for detecting direct ERα estrogenic interference (e.g., ERα binding, ERα-mediated nuclear mechanism) to a broad-spectrum analysis of ERα activation related to the mechanistic and functional outcomes of breast cancer cell proliferation.

## 2. Materials and Methods

Cell Culture and Reagents. 17β-estradiol (E2), DMEM (with and without phenol red), and fetal calf serum were purchased from MERCK (Darmstadt, Germany). Bradford protein assay kit, as well as anti-mouse and anti-rabbit secondary antibodies, was obtained from Bio-Rad (Hercules, CA, USA). Antibodies against ERα (H-C20, rabbit), pS2 (FL-84, rabbit), cyclin B1 (GNS1, mouse), cyclin D1 (H-295, rabbit), AKT (B-1, mouse), anti-phospho-ERK (E-4, mouse), and ERK1/2 (C-14, rabbit) were obtained from Santa Cruz Biotechnology (Santa Cruz, CA, USA); anti-phospho-ERα (Ser118, mouse) and anti-phospho-AKT (pAKT Ser473, 193H12, rabbit) antibodies were obtained from Cell Signaling; anti-vinculin (mouse) antibody was purchased from MERCK (Darmstadt, Germany). Chemiluminescence reagent for Western blotting was obtained from BioRad Laboratories (Hercules, CA, USA). ERα inhibitor endoxifen (End) was purchased from Tocris (Bristol, UK). Diethyl phthalate (DEP) was purchased from MERCK (Darmstadt, Germany). PolarScreen™ ERα Competitor Assay Kit, Green (A15882) was acquired from Thermo Scientific (Waltham, MA, USA). All the other products were from MERCK (Darmstadt, Germany). Analytical- or reagent-grade products were used without further purification. 

Human MCF-7 breast cancer cells were purchased from ATTC (LGC Standards S.r.l., Milano, Italy). MCF-7 cells were grown in air containing 5% CO2 in modified DMEM medium containing 10% (*v/v*) fetal calf serum, gentamicin (0.1 mg/mL), L-glutamine (2 mM), and pen–strep solution (penicillin 100 U/mL and streptomycin 100 mg/mL). The identity of the cell line was verified by STR analysis (BMR Genomics, Padova, Italy). When reported, cells were treated with 17β-estradiol (E2) and/or diethyl phthalate (DEP) at the indicated doses for the indicated periods; endoxifen (End) was added alone or 1 h before E2 and DEP administration and maintained throughout the reported time of stimulation.

In Vitro ERα Binding Assay. A fluorescence polarization (FP) assay was used to measure the binding affinity of diethyl phthalate (DEP) and 17β-estradiol (E2) for recombinant ERα in vitro. The FP assay was performed using a PolarScreen™ ERα Competitor Assay Kit, Green (A15882, Thermo Scientific), as previously reported [27]. Briefly, different doses (10^−12^–10^−5^ M) of DEP were administrated in the assay reaction containing Fluormone ES2 (4 nM) and ERα (75 nM) in binding buffer, and measurements were conducted in accordance with the instructions of the manufacturer. The assay was performed for each sample in quintuplicate for 2 h to reach steady-state conditions at room temperature in the dark, and results were read by using a Tecan Spark microplate reader for detecting fluorescence polarization [27]. 

Cell Manipulation for Western Blotting Analyses. Cells were grown in DMEM with phenol red plus 10% fetal calf serum for 24 h. Before stimulations, media were changed with DMEM without phenol red plus 1% of charcoal-stripped fetal calf serum, and then cells were treated with DEP and E2 at the indicated doses for the indicated periods in the presence or absence of End pre-treatment (10^−6^ M, 1 h before). After treatment, cells were lysed in Yoss Yarden (YY) buffer (50 mM Hepes (pH 7.5), 10% glycerol, 150 mM NaCl, 1% Triton X-100, 1 mM EDTA, and 1 mM EGTA) plus protease and phosphatase inhibitors. 

Western blot analysis was performed by loading 20–30 μg of protein on SDS gels. Gels were run, and the proteins were transferred to nitrocellulose membranes with a Turbo-Blot semidry transfer apparatus from Bio-Rad (Hercules, CA, USA). Immunoblotting was carried out by incubating the membranes with 5% milk or bovine serum albumin (60 min), followed by incubation overnight with anti-phospho-ERα (final dilution 1:1000), anti-ERα (final dilution 1:1000), anti-phospho-AKT (final dilution 1:1000), anti-AKT (final dilution 1:1000), anti-phospho-ERK (final dilution 1:1000), anti-ERK1/2 (final dilution 1:1000), anti-pS2 (final dilution 1:1000), anti-cyclin B1 (final dilution 1:1000), anti-cyclin D1 (final dilution 1:1000), and anti-vinculin (final dilution 1:40.000). Secondary antibody (final dilution 1:5000) incubation was continued for an additional 60 min. Bands were detected using a Chemidoc apparatus from Bio-Rad (Hercules, CA, USA).

Cell count. MCF-7 cells were grown in six-well plates in DMEM with phenol red plus 10% fetal calf serum for 24 h. Before stimulations, media were changed with DMEM without phenol red in the presence of 1% charcoal-stripped fetal calf serum, and cells were stimulated with DEP 10^−5^ M for 72 h in the presence or absence of End (10^−6^ M, 1 h before) pre-treatment. After stimulation, cells were harvested with trypsin, centrifuged, stained with trypan blue solution (0.5 mL; 0.4%), and counted in a hemocytometer (improved Neubauer chamber) in quadruplicate.

Cell Cycle Assays. Cell cycle analysis was performed using Nicoletti’s protocol [28]. Briefly, the cell pellet was resuspended in 500 μL of PBS, fixed by adding 4.5 mL of 70% cold ethanol, washed twice, and resuspended in 500 μL of PBS + 500 μL of DNA extraction buffer (0.19 M Na2HPO4, 0.004% Triton X-100, pH 7.8). Cells were incubated for 5 min at room temperature. The pellet was resuspended in 1 mL of DNA staining solution (20 μg of propidium iodide, 0.2 mg of RNase A, in PBS) and incubated once again for 30 min at room temperature. Finally, 20,000 total events on a linear scale were acquired with a CytoFlex Beckman Coulter (Brea, CA, USA), and the percentage of each cell cycle phase was calculated by a proper electronic marker.

Cellular DNA Content, Propidium Iodide (PI) Assay. MCF-7 cells were grown in 96-well plates in complete (10% fetal calf serum) DMEM with phenol red for 24 h. Media were replaced with DMEM without phenol red plus 1% charcoal-stripped fetal calf serum before cell treatment with DEP 10^−5^ M for 72 h in the presence or absence of End pre-treatment (10^−6^ M, 1 h before). The cells were fixed and permeabilized with frozen EtOH 70% for 15 min at −20 °C. EtOH solution was removed, and the cells were incubated with propidium iodide (PI) buffer for 30 min in the dark. The solution was removed, and the cells were rinsed with PBS solution. The fluorescence was revealed (excitation 537 nm, emission 621 nm) with a Spark 20 M multimode microplate reader.

Statistical Analysis. Densitometric analyses were performed using the free software Image J by quantifying the band intensity of the protein of interest with respect to the relative loading control band (i.e., vinculin) intensity. The statistical analysis was performed with ANOVA followed by the Bonferroni post-test using the InStat version 8 software system (Graph-Pad Software Inc., San Diego, CA, USA). In all cases, only values of *p* < 0.05 were considered significant.

## 3. Results

### 3.1. DEP Activates ERα without Binding the Receptor

**Figure 1:**
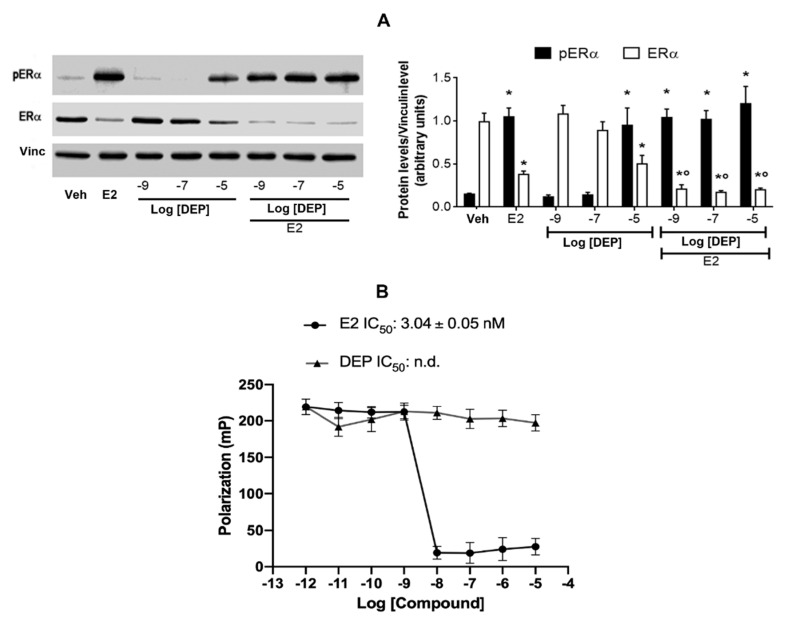
ERα activation and in vitro binding assays. Western blot (**left panel**) and corresponding densitometric analysis (**right panel**) of Ser118-phosphorylated ERα and total ERα protein level in MCF-7 treated for 1 h with the endogenous ERα ligand E2 (10^−8^ M) as positive control, and a dose curve of DEP at 10^−9^, 10^−7^, and 10^−5^ M in the presence or absence of E2 co-treatment (**A**). The vinculin levels were used as an internal control of protein loading. Data are means ± SD of at least four experiments. *p* < 0.01 was determined by the ANOVA test followed by the Bonferroni post-test vs. Veh (*) and E2 (°). In vitro ERα competitive binding assay for the endogenous ligand E2 and DEP performed over the 10^−12^–10^−5^ M range by using fluorescent E2 as a tracer. Inhibitor concentration (IC_50_- nM) is indicated in the panel for each compound (**B**).

One of the first mechanisms occurring after E2 binding to ERα is receptor activation throughout its phosphorylation at Serine 118 (Ser 118) followed by receptor degradation; these events are pivotal in ERα functional activation [29,30,31,32,33]. Consequently, to determine the estrogenicity of DEP, ductal carcinoma cells (MCF-7 cells) were treated for 1 h with increasing doses of DEP in the absence or presence of E2. Figure 1A shows that E2 stimulation (10^−8^ M) rapidly increased both receptor phosphorylation and degradation, while at just a 10^−5^ M concentration, DEP induced a similar effect. However, DEP co-treatment enhanced the E2-induced receptor degradation even at low doses (i.e., 10^−7^ and 10^−9^ M), increasing the receptor active status and suggesting an estrogen-agonistic role for this phthalate in the presence of ERα (Figure 1A).

The evaluation of ERα–ligand interaction is the most common assay performed to characterize the estrogenicity of EDCs [34], although divergent results have been reported in the literature on the ability of DEP to bind to ERα [18,19,35,36,37]. The in vitro polarization-based competitive binding assay of DEP and E2 to ERα was performed at room temperature and in steady-state conditions. Figure 1B shows that E2 displaced the fluorescent ligand (i.e., tracer) from ERα with an IC_50_ of about 3 nM comparable with the hormone K_d_ measured elsewhere [8,38]. On the other hand, DEP did not displace the fluorescent ligand over the range of 10^−12^–10^−5^ M, indicating that it does not bind to ERα in vitro (Figure 1B). Such evidence demonstrates that DEP could fully activate ERα without directly biding to it.

### 3.2. Analysis of DEP Effect on the Activation of Nuclear and Extra-Nuclear ERα Signals

**Figure 2:**
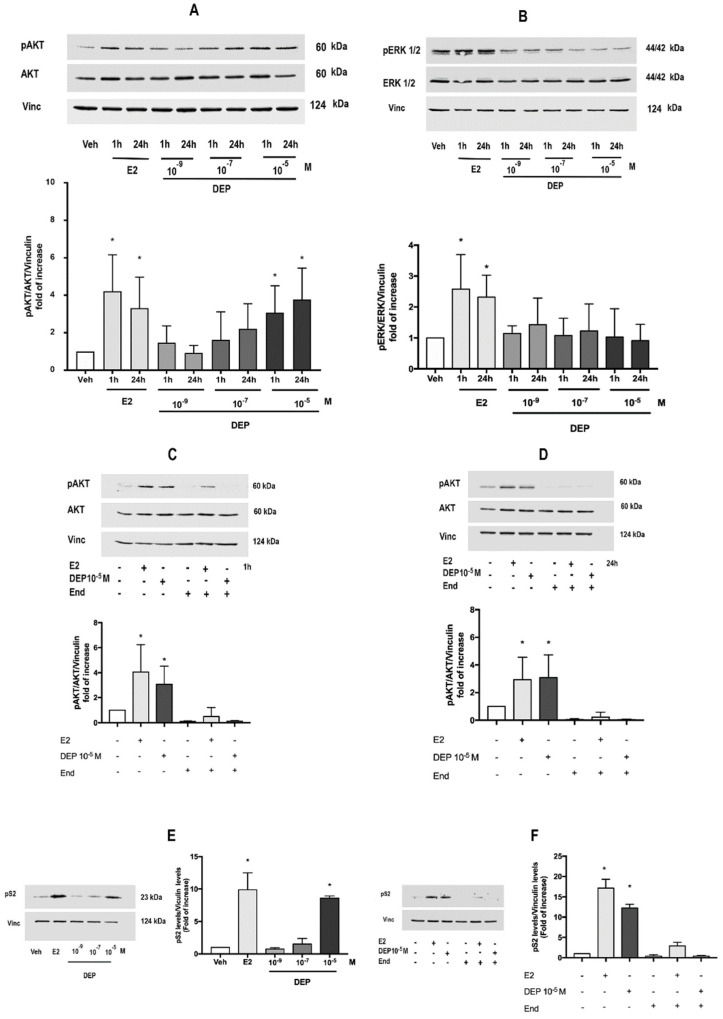
DEP activates nuclear and extra-nuclear rapid ERα signals. DEP dose-dependent effect on phosphorylation of AKT (**A**) and ERK1/2 (**B**) analyzed at short (1 h) and long (24 h) time exposures. Data show the Western blot image representative of at least four different experiments (**upper panel**) and the densitometric analysis (**bottom panel**). Western blot analysis of pAKT levels (**upper panel**) and densitometric analysis (**bottom panel**) in MCF-7 treated with DEP 10^−5^ M for 1 h (**C**) or 24 h (**D**) with or without cell pre-treatment with specific ERα inhibitor endoxifen (End; 10^−6^ M, 1 h before). Western blot (**left panel**) and corresponding densitometric analysis (**right panel**) of the E2-responsive protein pS2 in MCF-7 cells treated for 24 h with a dose curve of DEP (10^−9^, 10^−7^, 10^−5^ M) (**E**) or with DEP 10^−5^ M in the presence or absence of End (10^−6^ M, 1 h pre-treatment) (**F**). E2 in the presence and/or absence of End pre-treatment was used as internal positive control throughout all the experiments. The amount of protein was normalized in comparison with vinculin levels or with total AKT or total ERK1/2 and vinculin levels. Data are means ± SD of at least four experiments. *p* < 0.01 was determined by the ANOVA test followed by the Bonferroni post-test vs. Veh (-,-,-) (*).

ERα phosphorylation and degradation are critical events directly linked with the function of this receptor as a ligand-activated transcription factor; moreover, these mechanisms integrate the nuclear and extra-nuclear estrogen signals [1,9,29,32,39,40]. We then analyzed the downstream consequence of the DEP-induced E2-mimetic effect on receptor activation. In particular, the effect of DEP treatment on the main ERα-activated extra-nuclear cascade pathways pivotal for E2-induced cell proliferation (i.e., PI3K/AKT and ERK/MAPK) was evaluated at short-term (1 h) and long-term (24 h) exposures of diverse DEP concentrations. As previously reported [30,41], E2 treatment induced rapid (1 h) and persistent (24 h) ERK1/2 and AKT phosphorylation, whereas only DEP 10^−5^ M mimicked the rapid and persistent AKT activation (Figure 2 A) without any parallel effect on the ERK pathway activation (Figure 2B). Pre-treating MCF-7 cells with the ERα-specific inhibitor endoxifen (End, 10^−6^ M; 1 h pre-treatment) completely abolished the rapid (1 h, Figure 2C) and persistent (24 h, Figure 2D) effect of both E2 (10^−8^ M) and DEP (10^−5^ M) on AKT phosphorylation.

The impact of DEP on the direct nuclear action of ERα was evaluated by measuring the level of presenilin 2 (pS2), a protein encoded by an E2/ERα target gene whose promoter contains the canonical ERE sequence [29,39]. MCF-7 cell treatment with different doses of DEP ranging from 10^−9^ to 10^−5^ M for 24 h indicated that only at a 10^−5^ M concentration did DEP increase the pS2 level to a similar extent as that observed for the positive control E2 (10^−8^ M; 24 h) (Figure 2E). End pre-treatment (10^−6^ M) prevented the up-regulation of pS2 by both E2 and DEP (Figure 2F), supporting the notion that ERα is required for the DEP effect. Overall, these data demonstrate that, in line with the results obtained in Figure 1A on ERα phosphorylation and degradation, DEP (10^−5^ M) mimics the E2 effect, being able to activate ERα-dependent nuclear and extra-nuclear activities, showing a prevalent role of the PI3K/AKT pathway in the DEP effect. For this reason, 10^−5^ M of DEP was used for subsequent experiments.

### 3.3. DEP Induces Cyclin Expression and MCF-7 Proliferation

**Figure 3:**
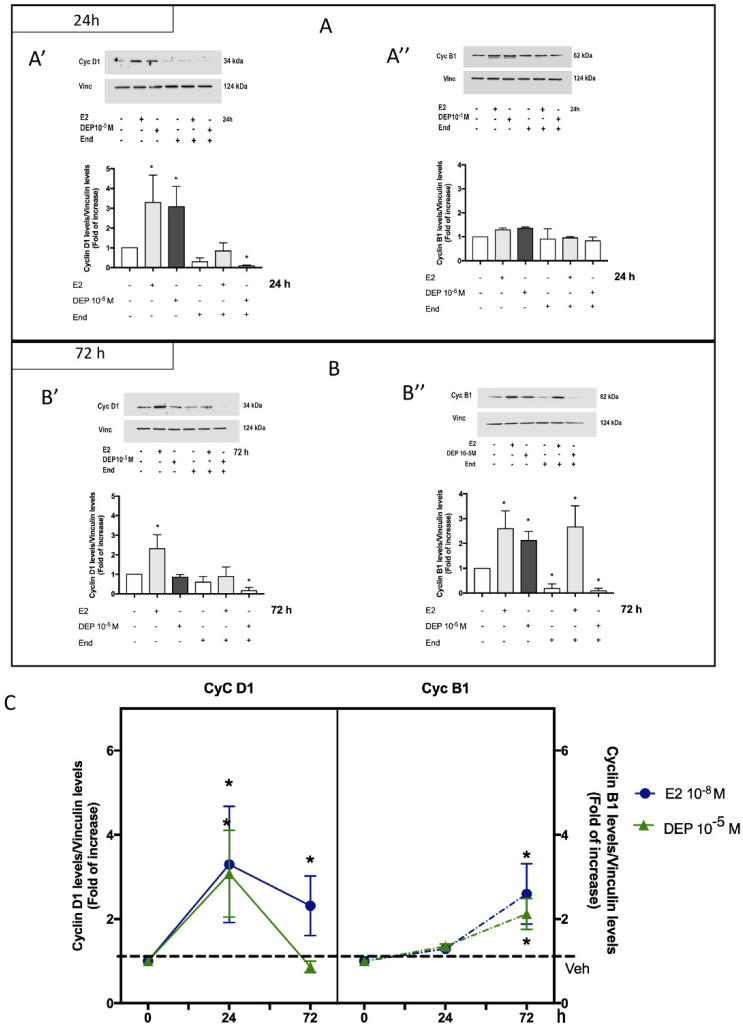
ERα-dependent effect of DEP on cyclin expression. MCF-7 cells were treated with DEP 10^−5^ M for 24 h (**A**) or 72 h (**B**) in the presence or absence of End (10^−6^ M, 1 h before) pre-stimulation, and the protein expression levels of cyclin D1 (Cyc D1; **A’**,**B’**) and cyclin B1 (Cyc B1; **A”**,**B”**) were evaluated by Western blot. Data show representative Western blot images of at least four experiments (**upper panels**) and the corresponding densitometric analysis (**bottom panels**). E2 with or without End pre-treatment was used as positive control. Analysis of expression of cyclin D1 (**left box**) and cyclin B1 (**right box**) as a function of the time of DEP 10^−5^ M exposure (**C**). The level of vinculin was used for protein normalization. Data are means ± SD of at least four experiments. *p* < 0.01 was determined by the ANOVA test followed by the Bonferroni post-test vs. Veh (-,-,-) (*).

It is well known that E2-dependent activation of ERα promotes DNA synthesis, cell cycle progression, and cell proliferation in estrogen-sensitive breast cancer cells (i.e., MCF-7) [6,42]. Prompted by the identification of DEP-dependent activation of ERα-based pathways important for E2-induced cell cycle progression (i.e., AKT activation), we investigated the DEP effect on cell proliferation-related intracellular events. As previously shown [41,43,44,45,46,47], cyclin D1 is under the indirect control of the E2/ERα pathway and is involved in the G1/S transition. Moreover, the cyclin B1 protein, which is necessary for G2/M passage, is also positively regulated by E2 in breast cancer cells [44]. Therefore, we analyzed the effect of DEP treatment at the 10^−5^ M concentration on the expression of both cyclins D1 and B1 at two different time points, 24 h and 72 h. The time of administration was chosen because it is known that the MCF-7 doubling time is 55 ± 11 h [48]. As reported in Figure 3A and in other reports [44], E2 (10^−8^ M) increased cyclin D1 but not cyclin B1 levels at the 24 h time point (Figure 3A). As with E2 treatment, DEP (10^−5^ M) stimulation did not modify the expression levels of cyclin B1 but significantly increased those of cyclin D1 at 24 h (Figure 3A). The same analysis conducted 72 h after the stimulation revealed that E2 up-regulated both cyclin D1 and cyclin B1 levels (Figure 3B), whereas DEP treatment promoted only the accumulation of cyclin B1 (Figure 3B”). The effect of DEP on cyclin D1 and cyclin B1 was completely abrogated by End (10^−6^ M; 1 h before) pre-treatment (Figure 3A’,B”), indicating an ERα-dependent mechanism. On the other hand, End pre-stimulation was able to block only the E2 effect on the modulation of cyclin D1 but not of cyclin B1, suggesting that E2 regulation of cyclin B1 could be more complex (e.g., the regulation of its expression could be dependent on the progression of the cell cycle). Furthermore, the fact that treatment with End alone or in combination with DEP significantly decreased the cyclin D1 (24 h, 72 h, Figure 3A’,B’) and cyclin B1 (72 h, Figure 3B”) levels significantly below the control conditions may support a critical role exerted by ERα in the regulation of the cell cycle and in the absence of any direct ligand/interactor. The reported results evidence that DEP exposure promoted the expression of critical E2-controlled regulators of the cell cycle through an ERα-dependent mechanism. This mechanism shows a “timing”, which was completely superimposable on that reported for E2 treatment with respect to cyclin B1 (i.e., increased levels at 72 h), whereas it did not completely coincide with respect to cyclin D1 levels (Figure 3C). Indeed, in the latter case, only E2 was able to maintain a persistent increase in the protein after 72 h; however, a decline in the protein level was evident beyond the 24 h time point (Figure 3C). 

**Figure 4:**
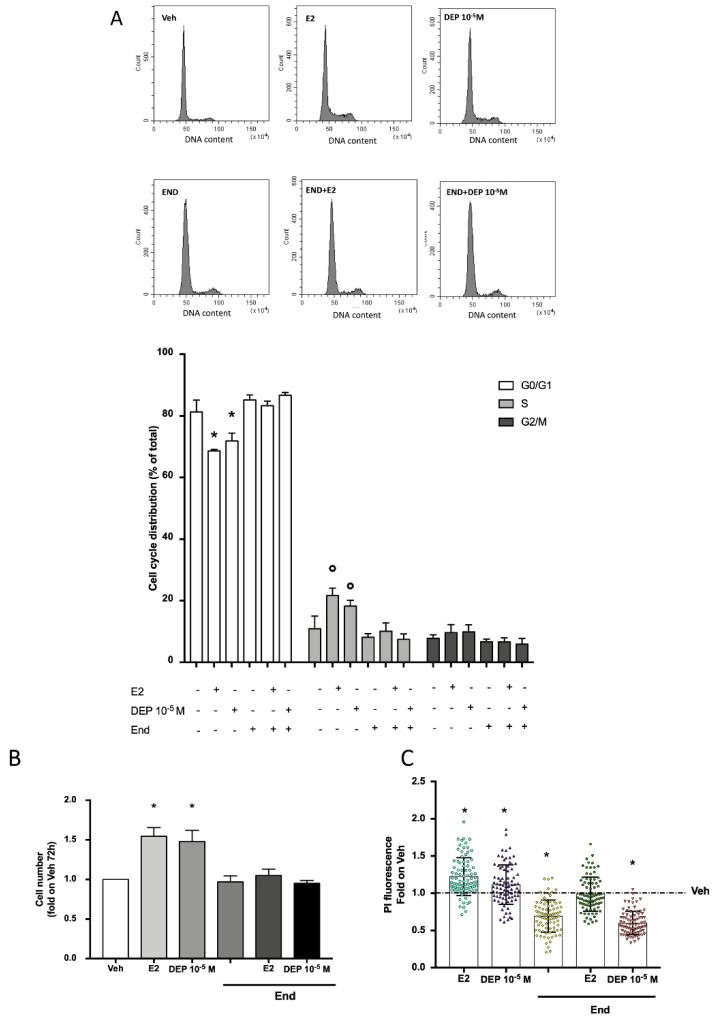
The effect of DEP on ERα-dependent DNA synthesis, cell cycle progression, and proliferation. Cell cycle analysis in MCF-7 cells treated with DEP 10^−5^ M for 24 h with or without pre-stimulation with End (10^−6^ M, 1 h before). **Upper panels** show flow cytometry analysis for cell cycle distribution of MCF-7 cells under different treatment conditions. The **bottom panel** shows the percentage of cells in G0/G1, S, and G2/M phases with respect to different stimulations. Histograms are representative of at least three independent experiments (**A**). Viable cell number (**B**) and analysis of total DNA content obtained from propidium iodide (PI) assay (**B**) of MCF-7 cells treated with DEP 10^−5^ M for 72 h in the presence or absence of End pre-treatment (10^−6^ M, 1 h before). Data are shown as fold of increase in Vehicle condition represented by the blank column (**B**) or as the dotted line (**C**). E2 stimulation with or without End pre-stimulation was used as positive control. *p* < 0.01 was determined by the ANOVA test followed by the Bonferroni post-test vs. G0/G1 phase Veh (-,-,-) and Veh (**B**,**C**) (*) or vs. S phase Veh (-,-,-) (°).

Once we defined the DEP-dependent increase in cyclins, we evaluated the impact of the phthalate on cell cycle progression at the same time of the maximum peak of cyclin D1 induction (24 h). E2 treatment significantly promoted an increased number of MCF-7 cells in the S phase of the cell cycle (Figure 4A). DEP treatment led to the same effect by promoting the G1-to-S phase transition of MCF-7 cells. To support the pivotal role of ERα in the DEP-dependent effect on the cell cycle, MCF-7 cells were pre-treated with End (10^−6^ M; 1 h before) which prevented the effect of both E2 and DEP. In turn, as reported in Figure 4B,C, the effect of DEP on cell cycle progression reflected the ability of the phthalate to significantly induce the proliferation of MCF-7 cells to the same extent as that of the endogenous ligand E2, as confirmed by viable cell counting (Figure 4B) and propidium iodide (PI) assay [49] (Figure 4C). As reported for the cell cycle progression, the pre-stimulation with End (10^−6^ M; 1 h before) blocked the proliferative effect of E2 and DEP (Figure 4B,C). These results demonstrate that DEP exposure fully activates the ERα nuclear and extra-nuclear signaling pathways devoted to breast cancer cell cycle progression and proliferation despite the lack of direct receptor binding.

## 4. Discussion

Phthalates comprise a wide range of chemicals grouped into high-molecular weight compounds (e.g., di-2-ethylexyl phthalate, DEHP) and low-molecular weight compounds (e.g., dibutyl phthalate, DBP) [50] which are environmentally widespread and have been identified as a human concern due to their endocrine disruption activities [35,50]. However, the mechanisms at the root of phthalate cellular functioning, concerning their estrogenic potential, are not completely elucidated and appear to be different when the compounds are used singularly or in a mixture [20]. Here, we considered the low-molecular weight diethyl phthalate (DEP) compound, which is not classified as estrogenic or as an anti-androgenic compound by European chemical agencies [51]. For the first time, we demonstrate that DEP acts as an estrogen mimetic able to indirectly activate ERα and, in turn, to increase the proliferation of human breast cancer cell models. These discoveries enlarge the idea about the mechanisms of estrogenic interference by chemical compounds and imply the need for the implementation of novel methods to confirm or to identify potential unknown endocrine disruptors. 

Phthalates including DBP and butyl benzyl phthalate (BBP) have been reported as week estrogen mimetics, being able to interact with ERα, induce receptor-dependent gene transcription, and increase the proliferation of MCF-7 breast cancer cells [16,25,52,53]. For a long time, evidence indicating no effect of DEP in the estrogen receptor transcriptional activation assay performed in yeast strains or in inducing augmentation of uterine weight in rats (uterotrophic assay) was related to the lack of agonistic effects of DEP on estrogen receptors and, in turn, of any estrogenic potential of such a phthalate [19,25]. Despite this, other independent studies have lately supported the estrogenic nature of DEP, as confirmed by in vitro transactivation assays in CHO and MCF-7 cells, the increased expression of the E2-susceptible vitellogenin (VTG) gene in zebrafish embryos, and the positive uterotrophic effect in immature female rats [18]. In this context of controversial evidence, the data reported here indicate that DEP up-regulates, in an ERα-dependent manner, the levels of the pS2 protein, whose expression is under the control of ERE sequences, indicating the ability of DEP to activate the direct transcriptional effect of the receptor. The same effect has already been reported, and it has been suggested to rely on the interaction between DEP and ERα by comparison with other phthalates [18]. However, we demonstrate, according to other previous works [19,35,36,37], that DEP does not directly bind ERα in vitro over the 10^−12^–10^−5^ M range of concentrations, indicating a more complex interference effect of DEP on ERα, which could occur at different levels of the receptor-dependent estrogenic signaling. Indeed, although EDC studies are mainly focused on the analysis of compounds’ interference in the classical direct nuclear ER mode of action [26], ERs are also localized at the plasma membrane, functioning as extrinsic receptors (for review, see [6]). Thus far, it is well known that ERα membrane localization is needed for the E2-dependent regulation of different cellular processes including cell migration, survival, and proliferation [54,55,56,57] occurring through the activation of the most conserved ERα-dependent extra-nuclear signaling pathways such as PI3K/AKT, ERK/MAPK, and p38/MAPK (for review, see [6]). We firstly demonstrate that DEP activates AKT signaling rapidly and persistently through ERα. Moreover, although the activation of the MAPK/ERK pathway by high DEP concentrations has been reported elsewhere [18], here, the obtained results indicate that DEP 10^−5^ M does not lead to ERK phosphorylation, differently to what occurs upon treatment with E2. This result supports the idea that the activation of estrogen receptor and its related signaling is a function of the molecule administered to the cell and of receptor conformational changes that the specific ligand can generate [6]. 

As previously reported in breast cancer cells [30], the rapid E2-dependent activation of the PI3K/AKT but not the ERK/MAPK pathway regulates Ser118 phosphorylation of ERα and its degradation, both events indicating the functional activation of the receptor [30,31,32]. ERα phosphorylation at the Ser118 residue represents the main integration link between extra-nuclear and nuclear E2-dependent receptor signaling, resulting in the ERα rapid signaling-dependent receptor protection from the proteolytic breakdown and full activation of target gene transcription [1,32,40,58]. Moreover, E2-dependent proteasomal ERα breakdown relates to the transcriptional activities of the receptor, whereas lysosomal-dependent degradation is needed for the E2-induced extracellular events linked to breast cancer cell proliferation [31]. In the context of EDC estrogenicity, the connection of ERα Ser118 phosphorylation/breakdown with the cellular consequences of endocrine disruptor exposure has been evidenced for bisphenol A (BPA), a prototype of xenoestrogen, and the plant-derived flavonoid naringenin (Nar) which binds to both ERα and ERβ with a similar affinity [5,7]. Both BPA and Nar bind to ERα and activate the receptor by triggering Ser118 phosphorylation and the direct transcriptional activity on ERE-containing genes. In addition, only BPA induced parallel receptor degradation and the induction of MCF-7 proliferation as well as the endogenous ligand E2 [7], indicating that mechanisms of ERα activation are strongly susceptible to external ligands and critically linked to the cellular outcomes.

The present results prove that, despite the lack of direct ERα binding, DEP also induces ERα phosphorylation and degradation, as it occurs upon E2 or BPA binding, and it conveys the same cellular outcome. Indeed, although some investigators indicated that DEP exposure does not lead to a proliferative effect on breast cancer cells [16], other evidence [18,23,24] supports our observation (Figure 4B,C), which reveals the ability of this phthalate to induce MCF-7 cell proliferation. Our data provide new insights about the molecular signature of DEP-dependent proliferation, which converges on the same effector proteins activated by the endogenous ligand E2. Indeed, DEP treatment triggers the up-regulation of two different proteins which take part in the regulation of the cell cycle under E2 stimulation, namely, cyclin D1 and cyclin B1 [41,44]. Cyclin D1 has been reported for a long time as an E2 target in breast cancer cells [41,43,44] which plays a key function in the hormone-dependent promotion of G1/S transition, as confirmed by the increase in cells in the S phase after hormone treatment. Despite the well-known transcriptional regulation of the cyclin D1 gene by E2 treatment in hormone-sensitive breast cancer cells [45,46,47], it does not present any ERE sequence in the promoter region [41,59], and it was clarified that hormone induction of cyclin D1 is under the control of a DNA binding-independent effect of ERα relying on the receptor membrane rapid signaling [41]. In parallel, cyclin B1, which promotes progression toward the M phase of the cell cycle [60], is also under the regulation of the E2/ERα-activated rapid ERK/MAPK and PI3K/AKT signals [44]. Thus, in line with the evidence that xenoestrogen could interfere with non-ERE-containing gene transcription [41], as well as cyclin D1 and B1, by regulating ERα membrane starting signals, we identified a novel DEP-activated circuit. Indeed, DEP-dependent activation of PI3K/AKT signals through ERα is accompanied by the receptor’s phosphorylation in Ser118 and its degradation, which converge to a time- and ERα-dependent increase in cyclins D1 and B1, resembling the effect provided by treatment with the endogenous ligand E2, as further evidenced by the DEP promotion of cell cycle progression. Altogether, the obtained results confirm the estrogenicity of DEP, providing the proof of principle that the DEP effect is strictly dependent on the presence of ERα and on the activation of rapid extra-nuclear signaling devoted to the increase in breast cancer cell proliferation, although DEP does not bind the receptor.

The absence of any physical DEP/ERα interaction has weakened the hypothesis of a direct effect of DEP on estrogen signaling for a long time. Some reports indicate that the main endocrine-disrupting function of DEP could be limited to the regulation of steroid biosynthesis, resulting in a greater production of E2 [20,35], which, in turn, could be responsible for the ER-based effect induced by DEP [35]. In the context of breast cancer cells, although playing a common role as a negative regulator of estrogen signaling, evidence indicates that the aryl hydrocarbon receptor (AhR)-activated pathway promotes intratumoral estrogen synthesis, as indicated by the induction of aromatase expression in MCF-7, T47D-1, and MDA-MB-231 breast cancer cells [61]. Thus, the reported ability of DEP to act as an AhR agonist [20] could sustain the hypothesis that DEP interference on estrogenic signaling in breast cancer cells may occur by stimulating cellular E2 synthesis. Nonetheless, results about the DEP-dependent rapid PI3K/AKT activation together with the differential activation of the ERK/MAPK pathway between E2 and DEP (Figure 2A,B) could not be reconciled with the idea of an E2-mediated mechanism. Instead, it may be thought that DEP impacts estrogenic signals by stimulating the engagement and the activation of ERα through an indirect mechanism involving other receptors, as it occurs for other EDCs [30], whose identification is still a matter of study in our laboratory. In this context, the ERα-46 and ERα-36 variants, both expressed in MCF-7 [62,63], could be considered as possible alternative receptors for DEP. Although the differential and broader ligand affinities of ERα variants [63,64] may sustain a possible accommodation of DEP, the biological functions associated with these variants do not completely fit with those reported for DEP. Indeed, the ERα-46 antagonistic effect on the proliferative function of full-length ERα [62] and ERα-36-dependent inhibition of transactivation and genomic estrogen signaling (e.g., pS2 expression) [63] may lead to ruling out an engagement of ERα-46 and ERα-36 by DEP, although further studies on this may be worthy. 

Furthermore, it has been suggested that phthalates can exert their disruptive effects on estrogen signaling through both ERα and G protein-coupled estrogen receptor (GPER-1) [65]. In spite of this, the lack of any evidence of direct interaction between DEP and GPER-1 and of convincing evidence supporting the role of GPER-1 in the estrogen-dependent response in vivo [66] weakens the hypothesis of the involvement of the GPER-1 receptor in DEP effects. 

## 5. Conclusions

In conclusion, the evidence reported here adds a higher level of complexity to the study of xenoestrogens. Indeed, the definition of the ERα-dependent pathway activated by DEP corroborates the idea that compounds able to interfere with the estrogenic signaling through an ER-mediated effect could not only function as receptor partners but also modulate the estrogenic signaling through the indirect induction of a conformational change in ERα and subsequent transduction mechanisms/functional outcomes. Such alternative mechanisms of estrogenic signaling activation, over time, have led to underestimating the potential estrogenic interference of environmental compounds that do not directly bind the receptor as well as DEP. Among the different methods applied to EDCs, assays that have pointed to the analysis of direct binding with sex steroid receptors, gene transcription, DNA, and co-regulator binding (e.g., yeast two-hybrid assay, transcription assay, report gene assay) are considered the basic tools for the screening of ER-based endocrine activity [12,34,67]. However, the use of pathway-based risk assessment which relies on the analysis of signaling pathways to evaluate the estrogenicity of environmental compounds has found increasing interest in the scientific community as a valuable option for cell/animal outcome-based evaluation, laying the groundwork for a paradigm shift, which should be necessarily set on reliable assays [34].

In this scenario, the evidence reported here reveals a circuitry where ERα Ser118 phosphorylation, degradation, and transduction pathways are linked to the final cellular outcome (e.g., proliferation) under different conditions including exposure to endogenous ligands, classical ER-binding xenoestrogens (e.g., BPA [30]), and other interfering compounds such as DEP, despite the absence of any physical binding with the receptor. In line with such evidence, analyses of ERα Ser118 phosphorylation and receptor breakdown could serve as critical endpoints and predictive assays for the screening of broader potential xenoestrogens, and for the evaluation of functional consequences of their exposure in the challenging perspective to shift the paradigm on EDC identification and reconsider the risk assessment for estrogenic interference beyond the common assays used in the regulatory context.

## Data Availability

The data presented in this study are available on request from the corresponding author.
